# Characterization of the microbiota and chemical properties of pork loins during dry aging

**DOI:** 10.1002/mbo3.1157

**Published:** 2021-01-07

**Authors:** Akihito Endo, Ryosuke Koizumi, Yozo Nakazawa, Yuh Shiwa, Shintaro Maeno, Yoshihiko Kido, Tomohiro Irisawa, Yoshiki Muramatsu, Kotaro Tada, Masao Yamazaki, Takao Myoda

**Affiliations:** ^1^ Department of Food, Aroma and Cosmetic Chemistry Faculty of Bioindustry Tokyo University of Agriculture Hokkaido Japan; ^2^ Department of Agricultural Innovation for Sustainability Faculty of Agriculture Tokyo University of Agriculture Kanagawa Japan; ^3^ Department of Molecular Microbiology Faculty of Life Sciences Tokyo University of Agriculture Tokyo Japan; ^4^ NODAI Genome Research Center Tokyo University of Agriculture Tokyo Japan; ^5^ Department of Bioproduction and Environment Engineering Faculty of Regional Environment Science Tokyo University of Agriculture Tokyo Japan

**Keywords:** aroma, *Debaryomyces*, dry‐aging pork, microbiota, proteolysis, *Pseudomonas*

## Abstract

Dry aging (DA) allows for the storage of meat without packaging at 0 to 3°C for several weeks. It enhances the production of pleasant flavors, tenderness, and juiciness in meat. Due to the long storage period and roles of indigenous microbiota in the maturation of several meat products, the microbiota of DA meat is of interest in terms of microbial contributions and food hygiene but has not yet been characterized in detail. This study identified the microbiota of pork loins during DA using culturing and culture‐independent meta‐16S rRNA gene sequencing and elucidated its characteristics. The amounts of free amino acids and profiles of aroma‐active compounds were also monitored by high‐performance liquid chromatography and gas chromatography, respectively. The meta‐16S rRNA gene sequencing revealed that *Pseudomonas* spp. generally dominated the microbiota throughout DA; however, the culturing analysis showed marked changes in the species composition during DA. *Acinetobacter* spp. were the second most dominant bacteria before DA in the culture‐independent analysis but became a minor population during DA. The cell numbers of yeasts showed an increased tendency during DA, and *Debaryomyces hansenii* was the only microorganism isolated from all meat samples throughout DA. Well‐known foodborne pathogens were not observed in two microbiota analyses. The amounts of free amino acids were increased by DA, and the number of aroma‐active compounds and their flavor dilution values markedly changed during DA. Most microbial isolates showed positive reactions with proteolytic and lipolytic activities, suggesting their contribution to tenderness and aroma production in DA meats.

## Graphical abstract

Microbiota on pork surface considerably changed during dry aging. Most microbial isolates showed positive reactions with proteolytic and lipolytic activities. The amounts of free amino acids were increased, and the number of aroma‐active compounds and their flavor dilution values markedly changed during this process.

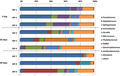

## 1 INTRODUCTION

The aging of meats is a well‐documented method in the meat industry that increases meat palatability by improving the tenderness, juiciness, and flavor of meat (Kim et al., 2018). Consumers have a favorable impression of aged meats and are willing to pay more for these products (Berger et al., 2018). This aging approach has been separated into two types based on the condition of aging: wet aging (WA) and dry aging (DA). WA involves keeping loins in a vacuumed package at −1 to 2°C for several weeks to several months (Kim et al., 2018). In contrast, DA involves the storage of loins without packaging at 0 to 3°C for several weeks. Product weight and trim losses are lower with WA than with DA. However, DA enhances the production of pleasant flavors, tenderness, and juiciness more than WA, resulting in consumer preference for DA meat (Berger et al., 2018; Kim et al., 2016, 2018; Lepper‐Blilie et al., 2016). These aging approaches are mainly applied to meats with low marbling levels, which are closely associated with the tenderness, flavor, and juiciness of meat (Corbin et al., 2015), but are also applied to highly marbled meats (Iida et al., 2016). Aging, particularly DA, is a promising approach to enhance marble‐associated sensory factors.

The preferable outcomes of aging described above are attributed to endogenous proteolysis during aging (Huff‐Lonergan & Lonergan, 2005; Kim et al., 2018; Maltin et al., 2003). However, the roles of the microbiota on the meat surface have been underestimated. Several proteolytic microorganisms have been identified on the surface of the meat (Liu et al., 2015; Wang et al., 2018). These microbes are regarded as risk factors for meat spoilage, whereas microbial proteolysis is a positive factor for meat fermentation (Ludemann et al., 2004; Nguyen et al., 2010). Several microbial proteases have been investigated for their ability to increase the tenderness of meat (Bolumar et al., 2005; Casquete et al., 2011; Sun et al., 2018). Several microbes, including yeast, molds, and bacteria, have been used as starter cultures to enhance flavor and safety and shorten ripening times in fermented meat products (Laranjo et al., 2019). The microbiota or starter microbes limit the adherence or growth of undesired microorganisms in the maturation of several meat products (Campanini et al., 1993; Laranjo et al., 2019; Simoncini et al., 2015). Furthermore, microbial lipolysis has been suggested to contribute to the aroma through the production of flavor compounds from lipids in meat (Pasini et al., 2018). Despite the potential importance of indigenous microbiota and their development on meats during aging, it has been poorly characterized. The characterization of microbiota will also contribute to the quality control and hygiene of aging meat.

In this study, moderately marbled pork loins were aged by drying because of the pleasant outcomes in DA as described above. The microbiota of DA loins was studied by culturing and meta‐16S rRNA gene sequencing. Changes in free amino acid compositions and aroma‐active compounds were monitored to characterize aging.

## 2 MATERIALS AND METHODS

### 2.1 Raw materials and the aging process

Three pork carcasses were obtained 3 days postmortem from 200‐ to 210‐day‐old LWD (three‐way cross‐bred Landrace x Large White x Duroc) pigs (*Sus scrofa domesticus*) that were mainly fed sweet potatoes, wheat, barley, corn, defatted soybeans, and fish powder. The carcasses, which were approx. 14 kg in weight and had marbling scores ranging between 3 and 4 based on the marbling quality standard by the National Pork Board (NPB, 2011), were divided into two skinless bone‐in loins each. One of the two loins obtained from each carcass was used as pre‐aging samples (day 0; samples named MP‐1, MP‐2, and MP‐3). The other part of the loins was assigned to DA without packaging, where the loin parts were placed at 1 to 2°C and relative humidity of 75 to 80% with fans (air velocity of 0.5 m/s) for 40 days. After 20 days of DA, loin samples were divided into two groups: Half‐aged samples (20‐day DA; samples named MP‐4, MP‐5, and MP‐6) and half loins stored for a further 20 days were used as full‐aged samples (40‐day DA; samples named MP‐7, MP‐8, and MP‐9). Pork loins were not treated with additives during aging.

### 2.2 Chemical analysis

#### 2.2.1 Measurement of pH, moisture, and surface water activity

The surfaces of loin samples were trimmed with a knife (thickness <1 cm), used to assess the water activity, and then subjected to a microbiota analysis. Water activity was assessed with AW Sprint TH500 (Novasina, Switzerland). Trimmed loin samples were minced with the food processor model OMF‐500 K (Ohmichi, Japan). The pH of minced loins was assessed with the pH meter model D‐51 (Horiba, Japan). Moisture content in the loins was evaluated by measuring the weight of minced pork before and after heating at 120°C with MX‐50 (A&D, Japan).

#### 2.2.2 Measurements of free amino acids and dipeptides

Minced loin samples (2.5 g) were diluted in 10 ml of 5% (w/v) sulfosalicylic acid and homogenized with T18 ULTRA‐TURRAX (IKA, Germany) for 1 min. Homogenized samples were centrifuged at 3000 rpm for 10 min, and the resultant supernatants were recovered. Pellets were re‐diluted with 10 ml of 5% sulfosalicylic acid, vortexed for 3 min, and centrifuged. The resultant supernatants were combined with the previously prepared supernatants and centrifuged at 10,000 rpm at 4°C for 10 min. Supernatants were adjusted to pH 2.2 with 1 M LiOH and prepared in a final volume of 100 ml with Milli‐Q water. They were then passed through a membrane filter (0.45 μm, Shimadzu, Japan) and analyzed with high‐performance liquid chromatography (HPLC) Amino Acid Analysis System (Shimadzu), according to the manufacturer's instructions.

#### 2.2.3 Aroma volatile analysis

To study the aroma volatiles in grilled meat, each meat sample was cut into 1‐cm‐thick slices, grilled on an electrical grill at 160–170°C for 2 min, and immediately homogenized with a hand mixer (Model MZ‐S101, Panasonic, Japan) at 4°C. Homogenized samples were stirred with 200 ml of dichloromethane for 1 h three times and dehydrated using anhydrous sodium sulfate. The resulting organic phase was subjected to solvent‐assisted flavor evaporation distillation to remove nonvolatile compounds. The distillates obtained were dried with anhydrous sodium sulfate, concentrated by atmospheric distillation with the Vigreux Column (35 × 0.6 cm i.d., Sigma, USA), and used in gas chromatography–olfactometry (GC‐O) analysis and for gas chromatography–mass spectroscopy (GC‐MS).

GC‐O was conducted with an Agilent 7890 GC (Agilent Technologies Japan Ltd) equipped with DB‐FFAP or DB‐5 (30 m × 0.32 mm i.d., the film thickness of 0.25 μm; Agilent Technologies Japan Ltd) to identify aroma‐active compounds in cooked meat as described previously (Abe et al., 2020). GC‐MS was performed using Agilent 7890A GC equipped with 5975 MSD (Agilent Technologies Japan) to identify aroma‐active compounds.

In the aroma extract dilution analysis, the aroma concentrates obtained by distillation with the Vigreux Column were diluted with dichloromethane starting from a dilution ratio of 1:1, continuing with 1:4, and concluding with 1:1024. Aroma‐active compounds were separated by GC‐O on a DB‐FFAP column and subsequently sniffed by three experienced panelists. The strengths of aroma‐active compounds were expressed using the flavor dilution (FD) factors. FD factors of the characteristic aroma‐active compounds were considered the greatest dilution factor at which these odors were detectable, with greater dilution factors making stronger contributions to meat aroma.

### 2.3 Microbiota analysis

#### 2.3.1 Culture‐dependent analysis

The trimmed surface of loin samples was diluted in 0.1% peptone saline solution and homogenized with BagMixer 400P (Interscience, France) for 1 min at a stroke rate of 8 times per second. The homogenized samples were serially diluted with 0.1% peptone saline solution and plated onto potato dextrose agar (PDA; Nissui, Japan) supplemented with 100 μg/ml chloramphenicol, nutrient agar (NA, Nissui) supplemented with 10 μg/ml cycloheximide, and MRS agar (Thermo Fisher Scientific, USA) supplemented with 10 μg/ml cycloheximide and 10 μg/ml sodium azide for isolating fungi, aerobic bacteria, and lactic acid bacteria (LAB), respectively. The media were incubated at 25°C (PDA) or 30°C (NA and MRS agar) for 2 days. Eight to ten colonies were selected based on colony morphology and color from each agar, inoculated onto PDA slant agar (for fungi) or into the nutrient broth (for aerobic bacteria) or MRS broth (for LAB), and incubated at the appropriate temperatures overnight. Bacterial isolates were maintained at −80°C in sterile half‐strength MRS broth supplemented with 20% (v/v) glycerol, and fungal isolates were kept at 4°C on PDA slant agar.

To identify bacterial isolates, the preparation of template DNA and the amplification and sequencing of the 16S rRNA gene were conducted using previously described methods (Endo & Okada, 2005). Approximately 600 bp of the 5’‐ends of the 16S rRNA gene sequences was used in BLAST analysis on GenBank. To identify fungal isolates, the preparation of template DNA was performed as described previously (Endo & Okada, 2005), and the amplification and sequencing of the full internal transcribed spacer (ITS) region were conducted as described elsewhere (Casimiro et al., 2004). Approximately 600 bp of the 5’‐ends of the ITS region sequences was used in BLAST analysis in GenBank.

#### 2.3.2 Microbiome analysis by culture‐independent meta‐16S rRNA gene sequencing

The microbiome of aging pork was further studied using meta‐16S rRNA gene sequencing of the bacterial DNA of meat samples. The homogenized loin surfaces described above were centrifuged (200 rpm, 10 min) to remove pork particles, and the resulting supernatant was used to isolate bacterial genomic DNA as described elsewhere (Endo & Okada, 2005). Isolated DNA was used as the template to generate a 16S rRNA metagenome library. The following primer pairs, forward, 5’‐TCGTCGGCAGCGTCAGATGTGTATAAGAGACAGCCTACGGGNGGCWGCAG‐3,’ and reverse, 5’‐GTCTCGTGGGCTCGGAGATGTGTATAAGAGACAGGGACTACHVGGGTWTCTAAT‐3’, were used to amplify the V3 and V4 regions of bacterial 16S rRNA genes, as described previously (Tian et al., 2015). The nucleotide sequences of the Illumina adapter overhang are underlined. A PCR amplicon library was generated following the Illumina 16S sample preparation guide (16S Sample Preparation Guide, 15044223; Illumina, USA). Library quality was assessed on an Agilent 2200 Tapestation (Agilent Technologies Japan). Libraries were sequenced as paired‐end 300‐bp reads using MiSeq (Illumina) according to the manufacturer's instructions. Sequence processing was performed according to the method described elsewhere (Zabat et al., 2018). Raw paired‐end FASTQ files were quality‐filtered, trimmed, de‐noised, and merged using DADA2 (Callahan et al., 2016) in QIIME2 (ver. 2018.11). During this process, the forward and reverse primer sequences were trimmed, and reads were truncated using the following parameters: ‐‐p‐trim‐left‐f 17, ‐‐p‐trim‐left‐r 21, ‐‐p‐trunc‐len‐f 250, and ‐‐p‐trunc‐len‐r 250. Chimeric sequences were identified and removed using the consensus method in DADA2. By using DADA2, sequences were clustered into operational taxonomic units (OTUs) at 100% identity. A taxonomic analysis of OTUs was performed using the QIIME2 q2‐feature‐classifier plugin with a pre‐trained naïve Bayes classifier on the SILVA 99% OTU database (version 128) trimmed to the V3‐V4 regions of the 16S rRNA gene (DeSantis et al., 2006).

### 2.4 Proteolytic and lipolytic activities of isolates

Pre‐cultured isolates were streaked onto casein agar medium and cultured at 30°C for 72 h. The formula for casein medium was described previously (Harkin et al., 2015). After culturing, plates were flooded with 1% (v/v) HCl. Proteolysis was considered to be positive when a clear zone formed around the colonies. Microbial lipolytic activities were assessed by the streaking of isolates onto Sprit Blue Agar (Becton Dickinson, Japan) supplemented with Tween 80 as a lipid source, according to the supplier's instructions. The plate was incubated at 30°C for 72 h, and lipolysis was considered to be positive when a clear zone formed around the colonies. All isolates (*n* = 134) were included in this study.

### 2.5 Statistical analysis

The Tukey–Kramer test was performed to compare free amino acid contents, water activities, pH, and moisture during DA using Bell Curve for Excel (Social Survey Research Information, Japan). The Kruskal–Wallis test was used to compare the bacterial cell numbers of each microbial group during DA using IBM SPSS Statistics for Windows (Version 26; Armonk, NY: IBM Corp.). Statistical analyses for alpha‐ and beta‐diversity metrics based on the results of meta‐16S rRNA gene sequencing were conducted through the QIIME 2 “core‐metrics‐phylogenetic” command using the following parameters: –p‐sampling‐depth 56663. A *p*‐value of 0.05 was considered to be significant.

## 3 RESULTS

### 3.1 Chemical analysis

Surface water activity and the moisture content of the trimmed pork significantly decreased during the first 20 days of DA (*p* < 0.05) but did not markedly change in the latter 20 days, while pH slightly increased during DA (Table 1).

**TABLE 1:** Water activity, pH, and moisture in DA pork loins

Days	Water activity	pH	Moisture (%)
0 day	0.985 ± 0.001^a^	5.56 ± 0.02^a^	71.9 ± 1.5^a^
20 days	0.960 ± 0.007^b^	5.77 ± 0.10^ab^	69.6 ± 2.3^a^
40 days	0.961 ± 0.002^b^	5.82 ± 0.07^b^	69.3 ± 2.3^a^

Results are expressed as mean ±standard deviation.

^abc^Different lowercase letters in the same column are significantly different (*p* < 0.05).

**FIGURE 1:**
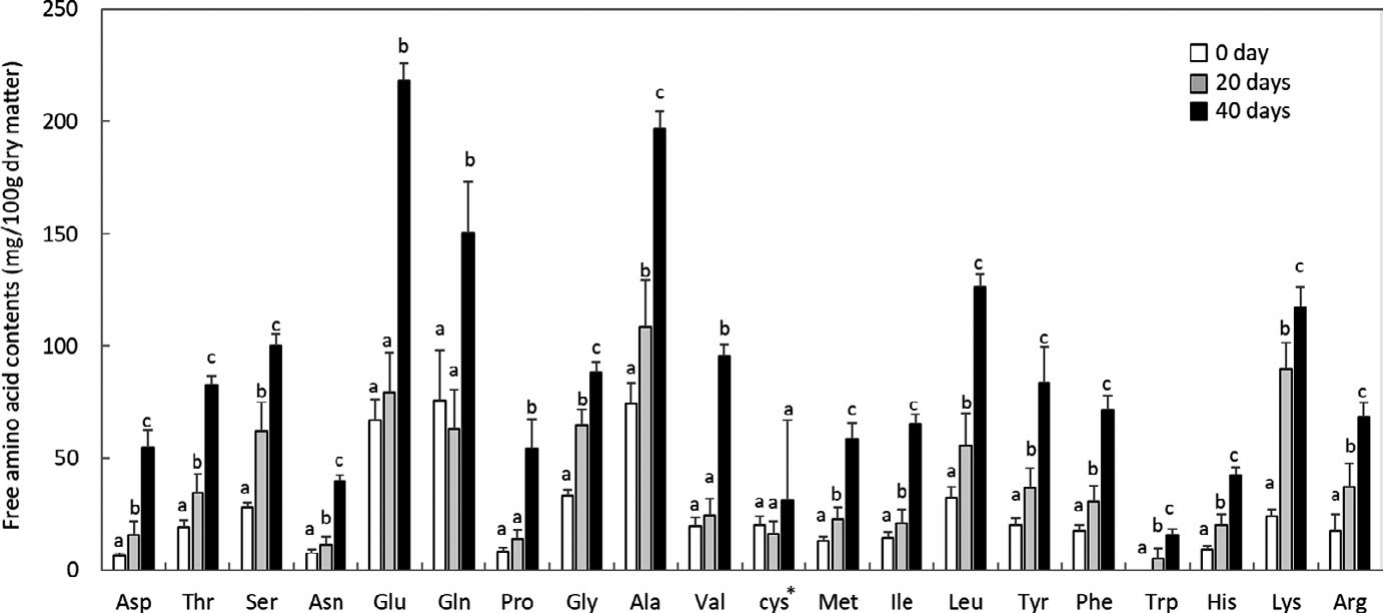
Changes in free amino acid contents during DA of pork loins. Different lowercase letters supplemented with bars are significantly different (*p* < 0.05). *detected as cystine in the system used (HPLC Amino Acid Analysis System, Shimadzu)

Among the free amino acids examined, the concentrations of 17 amino acids increased continuously during aging (Figure 1). The concentrations of glutamine, glutamic acid, proline, and valine were stable during the first 20 days of aging but increased in the latter 20 days. Cystine concentrations remained stable during the aging period. Total free amino acid concentrations on days 0, 20, and 40 were 511 ± 76, 814 ± 169, and 1761 ± 110 mg/100 g of dry matter of meat, respectively, and significantly increased during the first 20 days (*p* < 0.001) and the latter 20 days (*p* < 0.001). The concentration of di‐amino acid carnosine significantly decreased after 40 days (1735 ± 160 vs 1399 ± 190 mg/100 g of dry matter of meat, *p* = 0.008), whereas that of anserine did not (114 ± 11 vs 101 ± 13 mg/100 g of dry matter of meats, *p* = 0.21).

The GC‐O analysis detected 25, 48, and 53 compounds from grilled meat samples aged 0, 20, and 40 days, respectively (Appendix 1). Regarding grilled meat samples before DA (day 0), the highest FD value of 256 was obtained from 3‐hydroxy‐2‐methyl‐4‐pyrone (aroma description, cotton candy) and eugenol (clove), followed by 3‐hydroxy‐6‐methyl‐2(2H)‐pyranone (seasoning‐like), 3‐ethyl‐2,5‐dimethylpyrazine (potato), and vanillin (vanilla), each with FD factors of 64 (Table 2). Concerning grilled meat samples aged 20 days, 3‐ethyl‐2,5‐dimethylpyrazine and 3‐hydroxy‐2‐methyl‐4‐pyrone exhibited the highest FD value of 1024, followed by 3‐hydroxy‐6‐methyl‐2(2H)‐pyranone with FD of 256. 2‐Nonanone (green, oily), 2‐methoxy‐4‐vinylphenol (smoky), eugenol, and indole (animal) were detected as an FD factor of 64. 3‐Hydroxy‐2‐methyl‐4‐pyrone was still the highest FD (FD =1024) compound in grilled meat aged for 40 days, followed by 3‐hydroxy‐6‐methyl‐2(2H)‐pyranone and 2‐nonanone (FD =256). The FD values of eugenol and vanillin gradually decreased with aging. In contrast, the FD factors of 2‐methylpropanoic acid (cheesy), (E,E)‐2,4‐nonadienal (nutty, green), (E)‐6‐decenal (beans), γ‐decalactone (fruity, sweet), δ‐undecalactone (cosmetic), δ‐dodecalactone (peach), and 2‐phenylacetate (honey) were increased by DA.

**TABLE 2:** Aroma‐active compounds and their FD factors in grilled DA pork assessed by AEDA (extracted)

RI^a^		Aroma description	Compound	FD factor		
FFAP	DB−5			Day 0	Day 20	Day 40
1270	1203	Beans	(*E*)−6‐decenal	nd^b^	16	16
1373		Nutty, earthy	2‐ethyl−3‐methylpyrazine	nd	1	4
1400	1093	Green, oily	2‐nonanone	4	64	256
1419		Acid	2‐decanone	nd	1	4
1432	1079	Potato	3‐ethyl−2,5‐dimethylpyrazine	64	1024	16
1557	789	Cheesy	2‐methylpropanoic acid	16	16	64
1600	1251	Green, oily	(*Z*)−2‐decenal	nd	nd	1
1626		Green oily	(*E*)−2‐decenal	4	16	16
1700	1216	Nutty, green	(*E*,*E*)−2,4‐nonadienal	nd	16	64
1955		Cotton candy	3‐hydroxy−2‐methyl−4‐pyrone	256	1024	1024
2038	1047	Seasoning‐like	3‐hydroxy−6‐methyl−2(2H)‐pyranone	64	256	256
2146	1471	Fruity, sweet	γ‐decalactone	nd	4	4
2164	1359	Clove	Eugenol	256	64	1
2196	X	Smoky	2‐methoxy−4‐vinylphenol	16	64	64
2164	1359	Clove	Eugenol	256	64	1
2196	X	Smoky	2‐methoxy−4‐vinylphenol	16	64	64
2283	1606	Cosmetic	δ‐undecalactone	4	4	16
2300		Oily	α‐sinensal	nd	16	64
2380		Peach	δ‐dodecalactone	4	16	16
2556		Honey	2‐phenylacetate	nd	4	64
2569	1406	Vanilla	Vanillin	64	16	4

a Linear retention index on DB‐FFAP and DB‐5.

b Not detected.

### 3.2 Microbial composition determined by culturing

**FIGURE 2:**
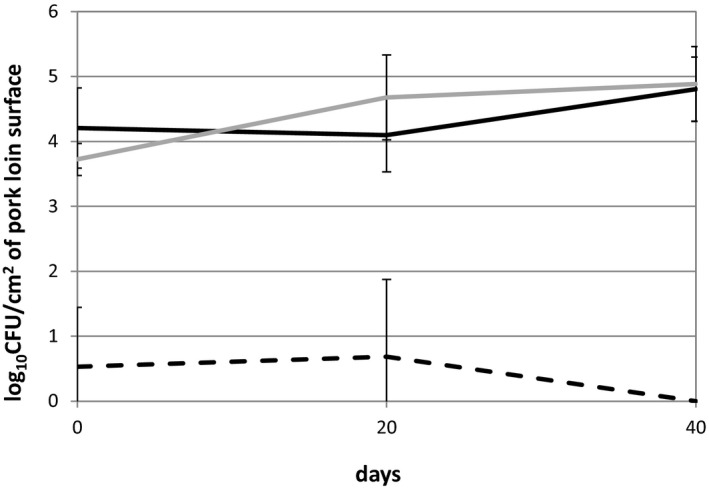
Viable cell numbers in DA pork loins on nutrient agar (black line), PDA agar (gray line), and MRS agar (dashed line). No significant differences (*p* > 0.05) were observed among the different time points

Viable cell counts on the surface of DA pork were assessed for aerobic bacteria, fungi, and LAB using NA, PDA, and MRS agar, respectively. The colony‐forming units (CFUs) of aerobes were stable during the initial 20 days of aging (4.21 and 4.10 log_10 _CFU/cm^2^ on days 0 and 20, respectively), but slightly increased to 4.80 during the latter 20 days (Figure 2). The CFU of yeast showed an increased tendency from 3.72 to 4.68 (*p* < 0.2) during the initial 20 days and to 4.88 by day 40. Molds were not recovered from samples. LAB were isolated from only one of the triplicated pork samples on days 0 and 20 at a low level (less than 1.00 log_10 _CFU/cm^2^) and were not detected in samples after 40 days of aging.

The 16S rRNA gene sequencing of bacterial isolates revealed that diverse *Pseudomonas* species dominated on the surface of pork loins before aging (day 0, Table 3). Due to the difficulties associated with identification because of high sequence similarities among several *Pseudomonas* species, they were described as phylogenetic groups in this study. Three of the six *Pseudomonas* species on day 0, that is, *Pseudomonas* groups A, C, and E, were observed in all three meat samples on day 0, while two of the remaining three species, that is, *Pseudomonas* group B and *Pseudomonas lurida*, were found in two of the three samples tested. However, none of these *Pseudomonas* species were recovered from DA samples on days 20 and 40. On day 20, *Pseudomonas* species were not observed in any multiple samples tested. At the end of aging, *Pseudomonas koreensis*, which was not observed in samples on day 0 or 20, was isolated from all samples tested. *Staphylococcus epidermidis* was found in all three samples tested on days 0 and 20 but disappeared on day 40. *Staphylococcus equorum* appeared on day 20 and was observed in two of the three samples tested on day 40. *Staphylococcus vitulinus* was also recovered from two of the three samples on day 40. Other species isolated were observed only in either the tested samples. The *Enterococcus devriesei* group was isolated as the sole LAB from one of the three samples on days 0 and 20. LAB were not recovered from samples on day 40.

**TABLE 3:** Microorganisms isolated from DA pork loins

	0 day	20 days	40 days
	*Pseudomonas* group A**	*Pseudomonas* group F	*Pseudomonas fluorescens*
	*Pseudomonas* group B*	*Pseudomonas* group G	Pseudomonas koreensis**
	*Pseudomonas* group C**	*Pseudomonas weihenstephanensis*	***Staphylococcus equorum*** *
	*Pseudomonas* group D	***Staphylococcus epidermidis*** **	Staphylococcus vitulinus*
	*Pseudomonas* group E**	***Staphylococcus equorum***	*Bacillus subtilis*
Aerobe	Pseudomonas lurida*	*Stenotrophomonas rhizophila*	*Rahnella variigena*
	***Staphylococcus epidermidis*** **	*Erwinia persicin‐aphidicola*	
	*Serratia liquefaciens*	*Macrococcus caseplyticus*	
	*Acinetobacter guillouiae*	*Acinetobacter septicus*	
	*Micrococcus aloeverae*		
	*Aeromonas salmonicida*		
LAB	***Enterococcus devriesei* group**	***Enterococcus devriesei* group**	ND.
Yeast	***Debaryomyces hansenii‐fabryi*** **	***Debaryomyces hansenii‐fabryi*** **	***Debaryomyces hansenii‐fabryi*** **
		Candida zeylanoides*	*Yarrowia deformans*
		***Yarrowia galli***	***Yarrowia galli***

Species isolated from triplicated and duplicated samples were shown with **and *, respectively. Species isolated at multiple time points were shown in bold.

*Pseudomonas* groups A, B, C, D, E, F, and G include *Pseudomonas paralactis/mucidolens/synxatha/gessardii/libanensis*, *Pseudomonas putida/fuscovaginae/aplenii*, *Pseudomonas marginalis/extremaustralis*, *Pseudomonas helleri/migulae*, *Pseudomonas lactis/cedrina*, *Pseudomonas fragi/psychrophila*, and *Pseudomonas oryzihabitans/reidholzensis*, respectively. The *Enterococcus devriesei* group includes *Enterococcus devriesei/pseudoavium/viikkiensis*.

*Debaryomyces hansenii* was isolated as the sole yeast from all three samples tested on day 0, and the organism was recovered from all samples throughout aging. *Candida zeylanoides* and *Yarrowia* spp. were also detected in samples aged 20 and 40 days.

### 3.3 Microbiome analysis by culture‐independent meta‐16S rRNA gene sequencing

**FIGURE 3:**
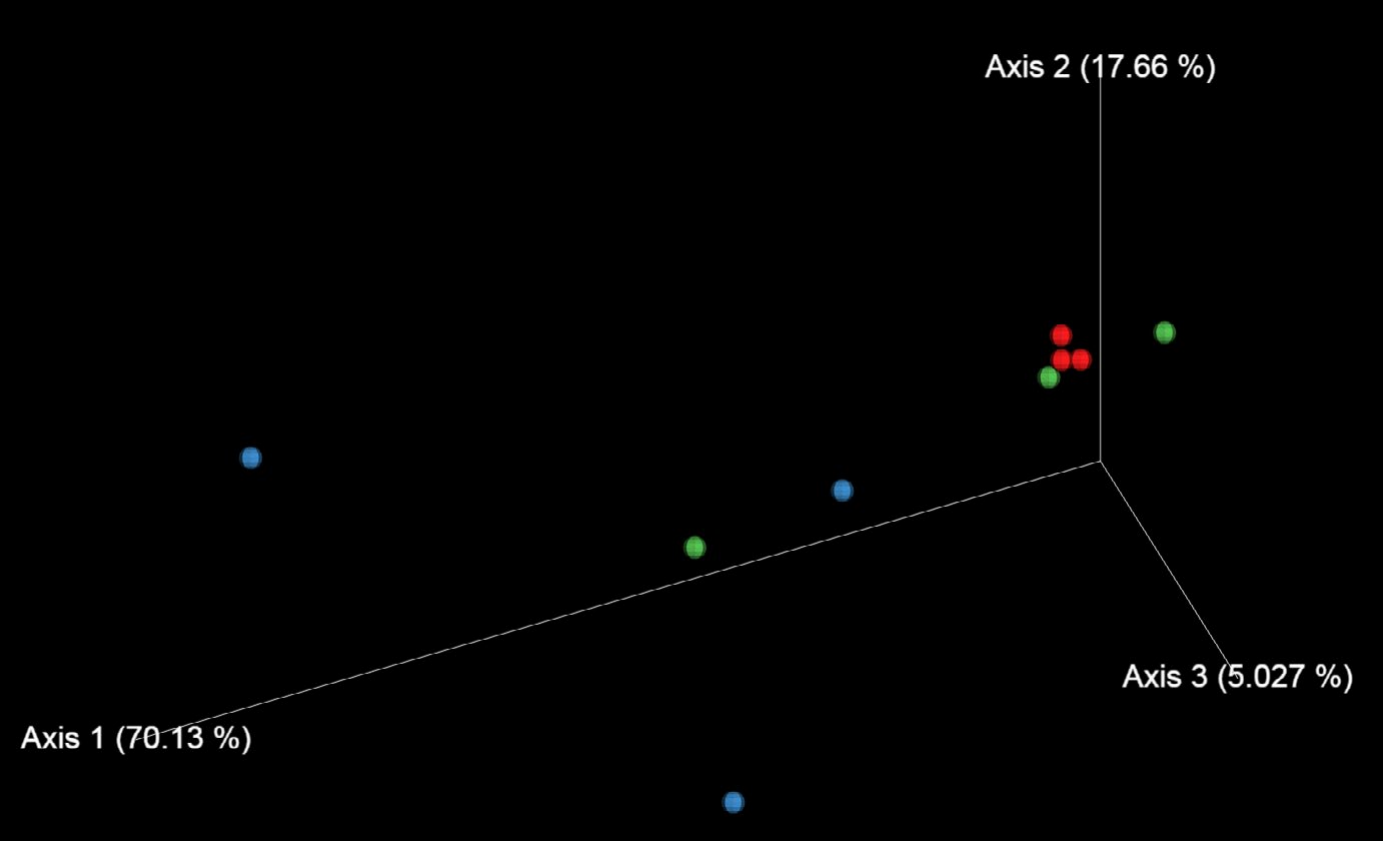
PCoA of 16S rRNA gene sequencing data at the genus level. Red, blue, and green symbols indicate data obtained from meat samples aged for 0, 20, and 40 days, respectively

Culture‐independent meta‐16S rRNA gene sequencing was also conducted to study changes in the microbiota on the surface of pork loins. The α‐diversity of the microbiota on the surface of DA pork revealed that microbial diversity significantly increased during the initial 20 days of aging (Table 4). The Shannon index indicated that diversity decreased during the latter 20 days of aging. The Evenness index revealed that species evenness decreased at the end of aging. The results of principal coordinate analysis (PCoA) showed that similar microbiota originally formed among samples before aging (day 0) (Figure 3). The microbiota markedly diversified after aging for 20 days; however, diversification subsided in the latter 20 days of aging.

**TABLE 4:** Diversity matrices of the microbiota on the surface of DA pork loins

	Day 0	Day 20	Day 40	*p* values		
				Day 0 vs day 20	Day 0 vs day 40	Day 20 vs day 40
Shannon index	4.97^a^	5.97	3.20	0.049	0.429	0.049
	(4.92, 5.06)	(5.59, 6.69)	(2.14, 5.45)			
Chao index	275	788	438	0.049	0.827	0.275
	(253, 588)	(633, 1275)	(124, 810)			
OTU number	275	755	434	0.049	0.827	0.127
	(253, 586)	(604, 1148)	(124, 732)			
Phylogenetic diversity	110	378	252	0.049	0.512	0.275
	(80, 222)	(276, 578)	(32, 391)			
Evenness index	0.62	0.65	0.45	0.275	0.127	0.049
	(0.54, 0.62)	(0.59, 0.66)	(0.37, 0.57)			

^a^ Median (minimum, maximum).

**FIGURE 4:**
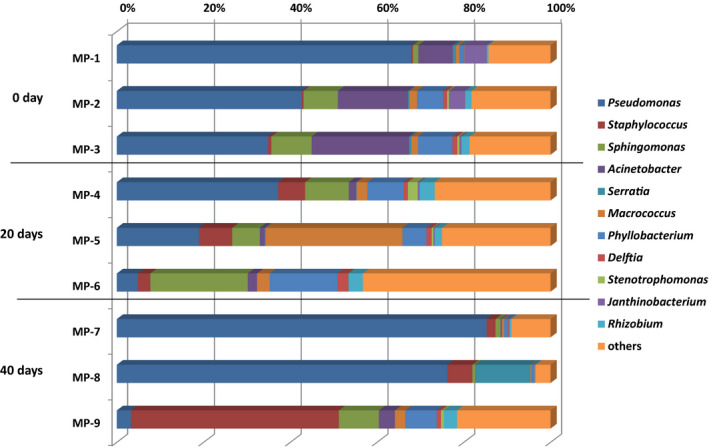
The relative abundance of each genus in pork loins with/without DA

The relative abundance of *Pseudomonas* accounted for more than 35% of all sequences recovered for each sample before aging (Figure 4), and *Acinetobacter* ranked as the 2nd major bacteria before aging. The relative abundance of *Pseudomonas* in each sample decreased to 4.9%−37.2% after the initial 20 days of aging but recovered to more than 75% in two of the three samples at the end of aging. The sample that had a low relative abundance of *Pseudomonas* (4.9%) on day 20 (MP‐6) continued to possess a low abundance of *Pseudomonas* (3.2%) on day 40 (MP‐9). *Acinetobacter* was a minor population after 20 days of aging. *Staphylococcus* was a minor population (less than 1%) before aging, but increased to 2.8–7.6 and 2.0%−48.0% after 20 and 40 days of aging, respectively.

### 3.4 Proteolytic and lipolytic activities in isolates

Most bacterial isolates exhibited proteolytic activities, whereas *Pseudomonas* group D isolates did not (Table 5). Strain‐dependent proteolysis was observed in *Pseudomonas* group C and *P. lurida*. In yeast isolates, *D. hansenii*, which was found in all meat samples tested, was negative for proteolysis. *Yarrowia* spp. exhibited proteolytic activities. Lipolytic activities were commonly observed in the isolates, while only some *D. hansenii* isolates were negative for this trait.

**TABLE 5:** Proteolytic and lipolytic activities found in isolates

Species	Proteolysis	Lipolysis
*Acinetobacter guillouiae*	+	+
*Acinetobacter septicus*	+	+
*Aeromonas salmonicida*	+	+
*Bacillus subtilis*	+	+
*Enterococcus devriesei group*	nd	nd
*Erwinia persicin‐aphidicola*	+	+
*Macrococcus caseplyticus*	+	+
*Micrococcus aloeverae*	+	+
*Pseudomonas* group A	+	+
*Pseudomonas* group B	+	+
*Pseudomonas* group C	v	+
*Pseudomonas* group D	‐	+
*Pseudomonas* group E	+	+
*Pseudomonas* group F	+	+
*Pseudomonas* group G	+	+
*Pseudomonas fluorescens*	+	+
*Pseudomonas koreensis*	+	+
*Pseudomonas lurida*	v	+
*Pseudomonas weihenstephanensis*	+	+
*Rahnella variigena*	+	+
*Serratia liquefaciens*	+	+
*Staphylococcus epidermidis*	+	+
*Staphylococcus equorum*	+	+
*Staphylococcus vitulinus*	+	+
*Stenotrophomonas rhizophila*	+	+
*Candida zeylanoides*	‐	+
*Debaryomyces hansenii*	‐	v
*Yarrowia deformans*	+	+
*Yarrowia galli*	+	+

Abbreviations: +, positive; ‐ negative; v, variable reactions; nd, not determined.

*Enterococcus devriesei* group isolates were not grown on the tested media to assess proteolysis and lipolysis.

## 4 DISCUSSION

DA is an aging approach that adds value to meat. DA keeps meat for more than 1 month, and this duration is sufficient for bacterial activities, even with storage at 1 to 2°C. Several microbes originating from meat products grow at low temperatures (Pothakos et al., 2014; Wickramasinghe et al., 2019). Microbial activities have a negative impact on changes in color, odor, and texture of fresh meat (Nychas et al., 2008); however, these negative effects have a lower impact on DA meats due to a trimming of the meat surface before consumption.

Culturing and culture‐independent meta‐16S rRNA gene sequencing techniques revealed that microbial compositions markedly changed during DA. Water activity on the surface of pork loins, the temperature used for DA, and the duration of storage may be among the key factors causing these changes. These factors are important for general microbial growth and have been reported to markedly affect the growth of meat‐origin *Pseudomonas* spp. (Lebert et al., 1998; Neumeyer et al., 1997). In this study, the water activity on the surface of pork loins decreased to 0.96 during the first 20 days of DA and remained stable until the end of DA. Water activity between 0.96 and 0.98 is generally the first bottleneck for microbial growth (Li & Torres, 1993; Sperber, 1983); however, certain microbes, including *Staphylococcus* spp. and fungi, grow with water activity less than 0.90 (Troller, 1986). These findings suggest that water activity of 0.96 in this study suppressed the growth of bacteria showing sensitivity to low water activity. Microbial cell numbers did not markedly increase on the surface of the meat during DA, whereas they increased by more than 1000‐fold on fresh meat during 9 days of storage at 4 °C (Li et al., 2019). Since storage temperatures were similar between the tests, different water activities would have prominent impacts on the whole microbial activities.

Culture‐independent meta‐16S rRNA gene sequencing confirmed that *Pseudomonas* spp. dominated the meat microbiota throughout DA; however, they were a minor population in MP‐6 and MP‐9 (Figure 4). These two samples were aged for 20 and 40 days, respectively, and originated from the same carcass. These results suggest that even when carcasses are prepared in the same manner and placed in a single refrigerator, the surface microbiota markedly changes among carcasses. *Sphingomonas* spp. were the major bacteria in MP‐6 (relative abundance of 22%) and *Staphylococcus* spp. in MP‐9 (relative abundance of 48%). *Staphylococcus* are resistant to osmotic stress (Valero et al., 2009). *Acinetobacter* spp. were the second major bacteria in the meat before DA but were a minor population after 20 or 40 days of DA. *Acinetobacter* spp. have been predominantly detected in short‐term stored fresh pork (Li et al., 2019) and have been reported to produce ropiness and brown diffusible pigments in beef (Vanderzant et al., 1987). Certain *Acinetobacter* spp. were previously shown to be resistant to desiccation stress (Zeidler & Muller, 2019), indicating the effects of other environmental factors, for example, temperature, pH, or microbial competition, on their activities. Well‐known foodborne pathogens, including *Salmonella* spp., *Listeria monocytogenes*, *Clostridium botulinum*, and diarrheagenic *Escherichia coli* (Fegan & Jenson, 2018), were not observed in culture‐dependent and culture‐independent analyses in this study, while certain pathogens, including *C. botulinum*, were not target organisms in the culture media used in this study.

At least 4 species of *Pseudomonas* were recovered from each meat loin sample before DA using the culturing technique; however, the numbers of *Pseudomonas* species isolated markedly decreased during DA and ranged between 0 and 3 and between 1 and 2 on days 20 and 40 of DA, respectively. *Pseudomonas* spp. are one of the major components of the microbiota of fresh meat and are regarded as one of the major spoilage microorganisms in fresh meat (Borch et al., 1996; Cauchie et al., 2019; Olofsson et al., 2007). However, meat surfaces are trimmed on DA meats before consumption, and thus, the dominant population is not a risk factor for spoilage. All *Pseudomonas* spp. found before DA were not recovered by culturing from meat samples after 20 and 40 days of aging. Other *Pseudomonas* spp. were observed in half‐ or full‐aged meat, whereas isolated *Pseudomonas* spp. differed between half‐ or full‐aged meat. The results obtained by culturing and meta‐16S rRNA gene sequencing suggest not only that *Pseudomonas* are generally dominant bacteria throughout DA, but also that their species composition markedly changes during aging. This may be the reason why the slightly decreased relative abundance of *Pseudomonas* on day 20 from that on day 0 increased by day 40 (Figure 4). *Pseudomonas koreensis* was isolated from all DA meat loin samples on day 40 but was not observed in samples on day 0 or day 20, suggesting better tolerance to osmotic stress than other *Pseudomonas* spp. This species is regarded as rhizosphere bacteria (Lopes et al., 2018), but has been recovered from chilled stored meats (Olofsson et al., 2007).

*Staphylococcus epidermidis* was isolated from all tested samples aged for 0 and 20 days (Table 3). The organism is a well‐known human skin commensal, implicating contamination from hands during meat processing. Similar findings were previously reported (Fraqueza et al., 2019). This species disappeared after 40 days of DA, while *S. equorum* and *S. vitulinus* were found in full‐aged meats. Since staphylococci are known to be osmotolerant, as described above, the different growth behaviors among species may be attributed to differences in tolerance to low temperatures.

The cell number of yeasts showed an increased tendency during DA. One of the reasons for this result may be their tolerance to osmotic stress (Hohmann et al., 2007). *Debaryomyces hansenii* was the sole yeast before DA and was recovered from all tested meat samples throughout DA. *Debaryomyces hansenii* has been identified as one of the major microbes in DA beef (Oh et al., 2019), and its beneficial properties, including the degradation of biogenic amines and production of volatile sulfur compounds in meat products, have been reported (Baumlisberger et al., 2015; Perea‐Sanz et al., 2019). Therefore, this organism is a starter candidate for several meat products (Murgia et al., 2019; Oh et al., 2019; Ramos et al., 2017), but not yet for DA pork. Collectively, the present results and previous findings suggest that this organism is a promising starter for DA pork. Molds, which have several roles in the maturation of meat products, but are also risk organisms for the production of mycotoxins (Merla et al., 2018), were not observed in this study.

The profiles of free amino acids and aroma‐active compounds markedly changed during DA. Proteolysis in DA meat is attributed to endogenous proteases (Huff‐Lonergan & Lonergan, 2005), whereas the mechanisms responsible for changes in aroma‐active components have not yet been characterized in detail. Proteolysis has been linked to meat tenderness (Kim et al., 2018). In this study, most bacterial and yeast isolates exhibited proteolytic activities. Therefore, they may contribute to the tenderness of DA meats, while the contribution may be less so than endogenous proteases. Proteolysis may also contribute to flavor development via the Maillard reaction through the accumulation of amino acids (Zhang et al., 2019). 3‐Hydroxy‐2‐methyl‐4‐pyrone, 2‐nonanone, and 3‐hydroxy‐6‐methyl‐2(2H)‐pyranone were detected as major aroma‐active compounds in full‐aged meats. Among these compounds, 3‐hydroxy‐2‐methyl‐4‐pyrone and 3‐hydroxy‐6‐methyl‐2(2H)‐pyranone are typically generated by the Maillard reaction. Moreover, several aroma‐active compounds continually increased (e.g., α‐sinensal, (*E,E*)‐2,4‐nonadienal, and 2‐phenylacetate) or decreased (e.g., eugenol and vanillin) during DA (Table 2). These compounds were not previously reported as characteristic compounds of aging (Ba et al., 2014; Watanabe et al., 2015). These differences may mainly be due to the different aging methods (DA vs. WA) and meats (pork vs. beef) used in the present and previous studies. Aliphatic aldehydes, ketones, and lactones detected in full‐aged meats, such as 2‐nonanone, (*E,E*)‐2,4‐nonadienal, δ‐undecalactone, and δ‐dodecalactone, may be produced by the decomposition and oxidation of lipids. While the oxidation of lipids in meats may occur naturally, microbes may also play a role. Further studies are needed to clarify the impact of microbiota on the meat quality of DA pork. This study was limited by the small sample size (*n* = 3); however, the results obtained provide important insights into microbial development and their potential roles in the palatability of DA pork.

## 5 CONCLUSION

The microbiota of pork loins markedly changed throughout DA, while changes in microbial cell numbers were smaller than those in fresh meat. Therefore, DA suppressed microbial activities at some level, whereas certain microbes showing tolerance to low temperature and low water activity maintained their activities. Several microbes observed in DA meat have been found in fresh meat, suggesting their origins. Even if the *Pseudomonas* genus dominated the microbiota, their species composition differed at the time points tested. The yeast *D. hansenii* was the sole microorganism found in all tested samples at different time points. Previous studies have reported the beneficial properties of this organism in the maturation/aging of meat products, and thus, it may be a promising starter candidate for stable aging. Well‐known foodborne pathogens were not observed in meat samples. Most microbial isolates exhibited both proteolytic and lipolytic activities. The amounts of free amino acids increased and the number of aroma‐active compounds and their contributions to the aroma were markedly changed by DA, which may have been due to endogenous and microbial enzymes.

## 6 ETHICS STATEMENT

None required.

## CONFLICT OF INTEREST

None declared.

## AUTHOR CONTRIBUTIONS

**Akihito Endo:** Conceptualization (lead); writing – original draft (lead); data curation (equal); formal analysis (equal); writing – original draft preparation (leading); writing – review and editing (lead); **Ryosuke Koizumi:** Conceptualization (supporting); data curation (equal); formal analysis (equal); methodology (equal); writing – review and editing (supporting); **Yozo Nakazawa:** Conceptualization (supporting); methodology (equal); writing – review and editing (supporting); **Yuh Shiwa:** data curation (equal); formal analysis (equal); writing – review and editing (supporting); **Shintaro Maeno:** data curation (equal); formal analysis (equal); writing – review and editing (supporting); **Yoshihiko Kido:** formal analysis (equal); writing – review and editing (supporting); **Tomohiro Irisawa:** formal analysis (equal); writing – review and editing (supporting); **Yoshiki Muramatsu:** Conceptualization (supporting); writing – review and editing (supporting); **Kotaro Tada:** formal analysis (equal); writing – review and editing (supporting); **Masao Yamazaki:** Conceptualization (supporting); formal analysis (equal); funding acquisition (leading); writing – review and editing (supporting); **Takao Myoda:** Conceptualization (supporting); data curation (equal); formal analysis (equal); methodology (equal); writing – review and editing (supporting).

## Data Availability

All data are provided in full in the results section of this paper apart from the 16S rRNA gene sequences of the isolates and meta‐16S rRNA sequencing data, which are available at NCBI GenBank under accession numbers LC532111–LC532139 and NCBI Sequence Read Archive under the accession number DRA011167, respectively.

## APPENDIX 1

### List of aroma‐active components in aging pork assessed by AEDA (full list)

**Table:**

RI*		Aroma description	Compounds	Aging periods		
FFAP	DB−5			Day 0	Day 20	Day 40
1078	800	Green	Hexanal	4	4	1
1108	940	Fruity	Ethyl−2‐methylpentanoate	16	1	16
1177		Fruity, floral	2‐methylpropyl−2‐methylbutanoate	nd**	1	4
1232	1025	Metal	α‐terpinene	nd	4	1
1245	1069	Mushroom	2‐(methylthio)heptane	4	4	16
1270	1203	Beans	(*E*)−6‐decenal	nd	16	16
1305		Nutty	2‐methyl−2‐thiazoline	nd	4	16
1341		Meaty	Octan−1‐thiol	nd	4	16
1373		Nutty, earthy	2‐ethyl−3‐methylpyrazine	nd	1	4
1400	1093	Green, oily	2‐nonanone	4	64	256
1405		Earthy	5‐ethyl−2,3‐dimethylpyrazine	1	1	1
1419		Acid	2‐decanone	nd	1	4
1432	1079	Potato	3‐ethyl−2,5‐dimethylpyrazine	64	1024	16
1450	905	Cooked potato	methional	nd	nd	4
1462	1122	Green	3‐nonen−5‐one	nd	4	1
1493	800	Cabbage	2‐mercaptopropan−1‐ol	16	16	1
1500		Coffee	5‐methyl−2‐furfurylthiol	4	1	1
1510		Acid	Unidentified	nd	1	1
1533		Oily	1‐(methylthio)nonane	nd	nd	1
1557	789	Cheesy	2‐methylpropanoic acid	16	16	64
1567		Camphorus	fenchol	nd	1	4
1592		Cheese	1‐phenylethanethiol	16	16	16
1600	1251	Green, oily	(*Z*)−2‐decenal	nd	nd	1
1626		Green oily	(*E*)−2‐decenal	4	16	16
1653		Gum	3‐mercaptopropan−1‐ol	nd	16	1
1663		Cheesy	Unidentified	nd	1	4
1700	1216	Nutty, green	(*E*,*E*)−2,4‐nonadienal	nd	16	64
1717		Cooked potato	methionol	nd	nd	1
1732		Nutty, roast	3‐mercapto−2‐methylpentan−1‐ol	16	4	64
1950		Meaty	Unidentified	Nd	4	1
1955		Cotton candy	3‐hydroxy−2‐methyl−4‐pyrone	256	1024	1024
2038	1047	Seasoning‐like	3‐hydroxy−6‐methyl−2(2H)‐pyranone	64	256	256
2069		Oily, soap	Octanoic acid	nd	4	1
2100	1328	Sweet	Unidentified	nd	4	4
2110		Sweet	Norfuraneol	16	4	64
2130	1058	Seasoning‐like	2‐mercaptopropanoic acid	64	4	1
2146	1471	Fruity, sweet	γ‐decalactone	nd	4	4
2164	1359	Clove	Eugenol	256	64	1
2194	1494	Fruity	δ‐decalactone	4	4	1
2196		Smoky	2‐methoxy−4‐vinylphenol	16	64	64
2214		Smoky, woody	Unidentified	nd	1	1
2222	1695	Steak	Decan−1,10‐dithiol	nd	nd	1
2250		Seasoning‐like	abhexone	nd	1	4
2283	1606	Cosmetic	δ‐undecalactone	4	4	16
2300		Oily	α‐sinensal	nd	16	64
2380		Peach	δ‐dodecalactone	4	16	16
2423		Soapy	Undecanoic acid	4	16	16
2453		Animal	Indole	nd	64	1
2469		Soap	Unidentified	16	4	1
2480		Green	Unidentified	nd	nd	1
2556		Honey	2‐phenylacetate	nd	4	64
2569	1406	Vanilla	Vanillin	64	16	4
>2600		Cinnamon like	Unidentified	4	4	4

* Linear retention index on DB‐FFAP and DB‐5.

** Not detected.

## References

[mbo31157-bib-0001] Abe, K. , Hori, Y. , & Myoda, T. (2020). Characterization of key aroma compounds in aged garlic extract. Food Chemistry, 312, 126081. 10.1016/j.foodchem.2019.126081 31901831

[mbo31157-bib-0002] Ba, H. V. , Park, K. , Dashmaa, D. , & Hwang, I. (2014). Effect of muscle type and vacuum chiller aging period on the chemical compositions, meat quality, sensory attributes and volatile compounds of Korean native cattle beef. Animal Science Journal, 85(2), 164–173. 10.1111/asj.12100 23911040

[mbo31157-bib-0003] Baumlisberger, M. , Moellecken, U. , Konig, H. , & Claus, H. (2015). The potential of the yeast debaryomyces hansenii H525 to degrade biogenic amines in food. Microorganisms, 3(4), 839–850. 10.3390/microorganisms3040839 27682120 PMC5023269

[mbo31157-bib-0004] Berger, J. , Kim, Y. H. B. , Legako, J. F. , Martini, S. , Lee, J. , Ebner, P. , & Zuelly, S. M. S. (2018). Dry‐aging improves meat quality attributes of grass‐fed beef loins. Meat Science, 145, 285–291. 10.1016/j.meatsci.2018.07.004 30007174

[mbo31157-bib-0005] Bolumar, T. , Sanz, Y. , Aristoy, M. C. , & Toldra, F. (2005). Protease B from Debaryomyces hansenii: purification and biochemical properties. International Journal of Food Microbiology, 98(2), 167–177. 10.1016/j.ijfoodmicro.2004.05.021 15681044

[mbo31157-bib-0006] Borch, E. , Kant‐Muermans, M. L. , & Blixt, Y. (1996). Bacterial spoilage of meat and cured meat products. International Journal of Food Microbiology, 33(1), 103–120. 10.1016/0168-1605(96)01135-x 8913812

[mbo31157-bib-0007] Callahan, B. J. , McMurdie, P. J. , Rosen, M. J. , Han, A. W. , Johnson, A. J. , & Holmes, S. P. (2016). DADA2: High‐resolution sample inference from Illumina amplicon data. Nature Methods, 13(7), 581–583. 10.1038/nmeth.3869 27214047 PMC4927377

[mbo31157-bib-0008] Campanini, M. , Pedrazzoni, I. , Barbuti, S. , & Baldini, P. (1993). Behaviour of Listeria monocytogenes during the maturation of naturally and artificially contaminated salami: effect of lactic‐acid bacteria starter cultures. International Journal of Food Microbiology, 20(3), 169–175. 10.1016/0168-1605(93)90109-t 8312141

[mbo31157-bib-0009] Casimiro, S. , Moura, M. , Ze‐Ze, L. , Tenreiro, R. , & Monteiro, A. A. (2004). Internal transcribed spacer 2 amplicon as a molecular marker for identification of Peronospora parasitica (crucifer downy mildew). Journal of Applied Microbiology, 96(3), 579–587. 10.1111/j.1365-2672.2004.02193.x 14962138

[mbo31157-bib-0010] Casquete, R. , Benito, M. J. , Martin, A. , Ruiz‐Moyano, S. , Cordoba, J. J. , & Cordoba, M. G. (2011). Role of an autochthonous starter culture and the protease EPg222 on the sensory and safety properties of a traditional Iberian dry‐fermented sausage "salchichon". Food Microbiology, 28(8), 1432–1440. 10.1016/j.fm.2011.07.004 21925025

[mbo31157-bib-0011] Cauchie, E. , Delhalle, L. , Taminiau, B. , Tahiri, A. , Korsak, N. , Burteau, S. , Fall, P. A. , Farnir, F. , Baré, G. , & Daube, G. (2019). Assessment of spoilage bacterial communities in food wrap and modified atmospheres‐packed minced pork meat samples by 16S rDNA metagenetic analysis. Frontiers in Microbiology, 10, 3074. 10.3389/fmicb.2019.03074 32038536 PMC6985204

[mbo31157-bib-0012] Corbin, C. H. , O'Quinn, T. G. , Garmyn, A. J. , Legako, J. F. , Hunt, M. R. , Dinh, T. T. N. , Rathmann, R. J. , Brooks, J. C. , & Miller, M. F. (2015). Sensory evaluation of tender beef strip loin steaks of varying marbling levels and quality treatments. Meat Science, 100, 24–31. 10.1016/j.meatsci.2014.09.009 25299587

[mbo31157-bib-0013] DeSantis, T. Z. , Hugenholtz, P. , Larsen, N. , Rojas, M. , Brodie, E. L. , Keller, K. , Huber, T. , Dalevi, D. , Hu, P. , & Andersen, G. L. (2006). Greengenes, a chimera‐checked 16S rRNA gene database and workbench compatible with ARB. Applied and Environment Microbiology, 72(7), 5069–5072. 10.1128/aem.03006-05 PMC1489311 16820507

[mbo31157-bib-0014] Endo, A. , & Okada, S. (2005). Monitoring the lactic acid bacterial diversity during shochu fermentation by PCR‐denaturing gradient gel electrophoresis. Journal of Bioscience and Bioengineering, 99(3), 216–221. 16233780 10.1263/jbb.99.216

[mbo31157-bib-0015] Fegan, N. , & Jenson, I. (2018). The role of meat in foodborne disease: Is there a coming revolution in risk assessment and management? Meat Science, 144, 22–29. 10.1016/j.meatsci.2018.04.018 29716760

[mbo31157-bib-0016] Fraqueza, M. J. , Rocha, J. M. , Laranjo, M. , Potes, M. E. , Fialho, A. R. , Fernandes, M. J. , Fernandes, M. H. , Barreto, A. , SemedoLemsaddek, T. , & Elias, M. (2019). What is the main processing factor influencing staphylococcus species diversity in different manufacturing units? Journal of Food Science, 84(10), 2932–2943. 10.1111/1750-3841.14796 31524954

[mbo31157-bib-0017] Harkin, C. , Bruck, W. M. , & Lynch, C. (2015). Isolation & identification of bacteria for the treatment of brown crab (Cancer pagurus) waste to produce chitinous material. Journal of Applied Microbiology, 118(4), 954–965. 10.1111/jam.12768 25644656

[mbo31157-bib-0018] Hohmann, S. , Krantz, M. , & Nordlander, B. (2007). Yeast osmoregulation. Methods in Enzymology, 428, 29–45. 10.1016/s0076-6879(07)28002-4 17875410

[mbo31157-bib-0019] Huff‐Lonergan, E. , & Lonergan, S. M. (2005). Mechanisms of water‐holding capacity of meat: The role of postmortem biochemical and structural changes. Meat Science, 71(1), 194–204. 10.1016/j.meatsci.2005.04.022 22064064

[mbo31157-bib-0020] Iida, F. , Miyazaki, Y. , Tsuyuki, R. , Kato, K. , Egusa, A. , Ogoshi, H. , & Nishimura, T. (2016). Changes in taste compounds, breaking properties, and sensory attributes during dry aging of beef from Japanese black cattle. Meat Science, 112, 46–51. 10.1016/j.meatsci.2015.10.015 26519608

[mbo31157-bib-0021] Kim, Y. H. , Kemp, R. , & Samuelsson, L. M. (2016). Effects of dry‐aging on meat quality attributes and metabolite profiles of beef loins. Meat Science, 111, 168–176. 10.1016/j.meatsci.2015.09.008 26437054

[mbo31157-bib-0022] Kim, Y. H. B. , Ma, D. , Setyabrata, D. , Farouk, M. M. , Lonergan, S. M. , Huff‐Lonergan, E. , & Hunt, M. C. (2018). Understanding postmortem biochemical processes and post‐harvest aging factors to develop novel smart‐aging strategies. Meat Science, 144, 74–90. 10.1016/j.meatsci.2018.04.031 29731371

[mbo31157-bib-0023] Laranjo, M. , Potes, M. E. , & Elias, M. (2019). Role of starter cultures on the safety of fermented meat products. Frontiers in Microbiology, 10, 853. 10.3389/fmicb.2019.00853 31133993 PMC6524729

[mbo31157-bib-0024] Lebert, I. , Begot, C. , & Lebert, A. (1998). Growth of Pseudomonas fluorescens and Pseudomonas fragi in a meat medium as affected by pH (5.8‐7.0), water activity (0.97‐1.00) and temperature (7–25 degrees C). International Journal of Food Microbiology, 39(1–2), 53–60. 10.1016/s0168-1605(97)00116-5 9562876

[mbo31157-bib-0025] Lepper‐Blilie, A. N. , Berg, E. P. , Buchanan, D. S. , & Berg, P. T. (2016). Effects of post‐mortem aging time and type of aging on palatability of low marbled beef loins. Meat Science, 112, 63–68. 10.1016/j.meatsci.2015.10.017 26551359

[mbo31157-bib-0026] Li, K. Y. , & Torres, J. A. (1993). Water activity relationships for selected mesophiles and psychrotrophs at refrigeration temperature. Journal of Food Protection, 56(7), 612–615. 10.4315/0362-028x-56.7.612 31113039

[mbo31157-bib-0027] Li, N. , Zhang, Y. , Wu, Q. , Gu, Q. , Chen, M. , Zhang, Y. , Sun, X. , & Zhang, J. (2019). High‐throughput sequencing analysis of bacterial community composition and quality characteristics in refrigerated pork during storage. Food Microbiology, 83, 86–94. 10.1016/j.fm.2019.04.013 31202422

[mbo31157-bib-0028] Liu, Y. J. , Xie, J. , Zhao, L. J. , Qian, Y. F. , Zhao, Y. , & Liu, X. (2015). Biofilm formation characteristics of pseudomonas lundensis isolated from meat. Journal of Food Science, 80(12), M2904–2910. 10.1111/1750-3841.13142 26551486

[mbo31157-bib-0029] Lopes, L. D. , Pereira, E. S. M. C. , Weisberg, A. J. , Davis, E. W. 2nd , Yan, Q. , Varize, C. S. , Chih‐Feng, W. U. , Chang, J. H. , Loper, J. E. , & Andreote, F. D. (2018). Genome variations between rhizosphere and bulk soil ecotypes of a Pseudomonas koreensis population. Environmental Microbiology, 20(12), 4401–4414. 10.1111/1462-2920.14363 30033663

[mbo31157-bib-0030] Ludemann, V. , Pose, G. , Pollio, M. L. , & Segura, J. (2004). Determination of growth characteristics and lipolytic and proteolytic activities of Penicillium strains isolated from Argentinean salami. International Journal of Food Microbiology, 96(1), 13–18. 10.1016/j.ijfoodmicro.2004.03.003 15358501

[mbo31157-bib-0031] Maltin, C. , Balcerzak, D. , Tilley, R. , & Delday, M. (2003). Determinants of meat quality: Tenderness. The Proceedings of the Nutrition Society, 62(2), 337–347. 10.1079/pns2003248 14506881

[mbo31157-bib-0032] Merla, C. , Andreoli, G. , Garino, C. , Vicari, N. , Tosi, G. , Guglielminetti, M. L. , Moretti, A. , Biancardi, A. , Arlorio, M. , & Fabbi, M. (2018). Monitoring of ochratoxin A and ochratoxin‐producing fungi in traditional salami manufactured in Northern Italy. Mycotoxin Research, 34(2), 107–116. 10.1007/s12550-017-0305-y 29299825

[mbo31157-bib-0033] Murgia, M. A. , Marongiu, A. , Aponte, M. , Blaiotta, G. , Deiana, P. , & Mangia, N. P. (2019). Impact of a selected Debaryomyces hansenii strain's inoculation on the quality of Sardinian fermented sausages. Food Research International (Ottawa, Ont.), 121, 144–150. 10.1016/j.foodres.2019.03.042 31108735

[mbo31157-bib-0034] Neumeyer, K. , Ross, T. , & McMeekin, T. A. (1997). Development of a predictive model to describe the effects of temperature and water activity on the growth of spoilage pseudomonads. International Journal of Food Microbiology, 38(1), 45–54. 10.1016/s0168-1605(97)00089-5 9498136

[mbo31157-bib-0035] Nguyen, H. T. , Elegado, F. B. , Librojo‐Basilio, N. T. , Mabesa, R. C. , & Dizon, E. I. (2010). Isolation and characterisation of selected lactic acid bacteria for improved processing of Nem chua, a traditional fermented meat from Vietnam. Beneficial Microbes, 1(1), 67–74. 10.3920/bm2009.0001 21831751

[mbo31157-bib-0036] NPB (2011). Official color & marbling quality standards. National Pork Board.

[mbo31157-bib-0037] Nychas, G. J. , Skandamis, P. N. , Tassou, C. C. , & Koutsoumanis, K. P. (2008). Meat spoilage during distribution. Meat Science, 78(1–2), 77–89. 10.1016/j.meatsci.2007.06.020 22062098

[mbo31157-bib-0038] Oh, H. , Lee, H. J. , Lee, J. , Jo, C. , & Yoon, Y. (2019). Identification of microorganisms associated with the quality improvement of dry‐aged beef through microbiome analysis and DNA sequencing, and evaluation of their effects on beef quality. Journal of Food Science, 84(10), 2944–2954. 10.1111/1750-3841.14813 31553057

[mbo31157-bib-0039] Olofsson, T. C. , Ahrne, S. , & Molin, G. (2007). Composition of the bacterial population of refrigerated beef, identified with direct 16S rRNA gene analysis and pure culture technique. International Journal of Food Microbiology, 118(3), 233–240. 10.1016/j.ijfoodmicro.2007.07.017 17765348

[mbo31157-bib-0040] Pasini, F. , Soglia, F. , Petracci, M. , Caboni, M. F. , Marziali, S. , Montanari, C. , Gardini, F. , Grazia, L. , & Tabanelli, G. (2018). Effect of fermentation with different lactic acid bacteria starter cultures on biogenic amine content and ripening patterns in dry fermented sausages. Nutrients, 10(10), 10.3390/nu10101497 PMC6213744 30322117

[mbo31157-bib-0041] Perea‐Sanz, L. , Peris, D. , Belloch, C. , & Flores, M. (2019). Debaryomyces hansenii metabolism of sulfur amino acids as precursors of volatile sulfur compounds of interest in meat products. Journal of Agricultural and Food Chemistry, 67(33), 9335–9343. 10.1021/acs.jafc.9b03361 31343169

[mbo31157-bib-0042] Pothakos, V. , Snauwaert, C. , De Vos, P. , Huys, G. , & Devlieghere, F. (2014). Psychrotrophic members of *Leuconostoc gasicomitatum*, *Leuconostoc gelidum* and *Lactococcus piscium* dominate at the end of shelf‐life in packaged and chilled‐stored food products in Belgium. Food Microbiology, 39, 61–67. 10.1016/j.fm.2013.11.005 24387853

[mbo31157-bib-0043] Ramos, J. , Melero, Y. , Ramos‐Moreno, L. , MichAn, C. , & Cabezas, L. (2017). Debaryomyces hansenii strains from valle de los pedroches iberian dry meat products: Isolation, identification, characterization, and selection for starter cultures. Journal of Microbiology and Biotechnology, 27(9), 1576–1585. 10.4014/jmb.1704.04045 28683529

[mbo31157-bib-0044] Simoncini, N. , Pinna, A. , Toscani, T. , & Virgili, R. (2015). Effect of added autochthonous yeasts on the volatile compounds of dry‐cured hams. International Journal of Food Microbiology, 212, 25–33. 10.1016/j.ijfoodmicro.2015.06.024 26210478

[mbo31157-bib-0045] Sperber, W. H. (1983). Influence of water activity on foodborne bacteria ‐ A review (1). Journal of Food Protection, 46(2), 142–150. 10.4315/0362-028x-46.2.142 30913607

[mbo31157-bib-0046] Sun, Q. , Chen, F. , Geng, F. , Luo, Y. , Gong, S. , & Jiang, Z. (2018). A novel aspartic protease from Rhizomucor miehei expressed in Pichia pastoris and its application on meat tenderization and preparation of turtle peptides. Food Chemistry, 245, 570–577. 10.1016/j.foodchem.2017.10.113 29287411

[mbo31157-bib-0047] Tian, B. Y. , Cao, Y. , & Zhang, K. Q. (2015). Metagenomic insights into communities, functions of endophytes, and their associates with infection by root‐knot nematode, Meloidogyne incognita, in tomato roots. Scientific Reports, 5, 17087. 10.1038/srep17087 26603211 PMC4658523

[mbo31157-bib-0048] Troller, J. A. (1986). Water relations of foodborne bacterial pathogens ‐ An updated review. Journal of Food Protection, 49(8), 656–670. 10.4315/0362-028x-49.8.656 30959698

[mbo31157-bib-0049] Valero, A. , Perez‐Rodriguez, F. , Carrasco, E. , Fuentes‐Alventosa, J. M. , Garcia‐Gimeno, R. M. , & Zurera, G. (2009). Modelling the growth boundaries of Staphylococcus aureus: Effect of temperature, pH and water activity. International Journal of Food Microbiology, 133(1–2), 186–194. 10.1016/j.ijfoodmicro.2009.05.023 19523705

[mbo31157-bib-0050] Vanderzant, C. , Savell, J. W. , Hamby, P. L. , Acuff, G. R. , Cox, N. A. , & Bailey, J. S. (1987). Indole‐lnduced, green to brown‐black pigment formation by an acinetobacter strain from beef. Journal of Food Protection, 50(6), 485–486. 10.4315/0362-028x-50.6.485 30965443

[mbo31157-bib-0051] Wang, G. , Ma, F. , Zeng, L. , Bai, Y. , Wang, H. , Xu, X. , & Zhou, G. (2018). Modified atmosphere packaging decreased Pseudomonas fragi cell metabolism and extracellular proteolytic activities on meat. Food Microbiology, 76, 443–449. 10.1016/j.fm.2018.07.007 30166172

[mbo31157-bib-0052] Watanabe, A. , Kamada, G. , Imanari, M. , Shiba, N. , Yonai, M. , & Muramoto, T. (2015). Effect of aging on volatile compounds in cooked beef. Meat Science, 107, 12–19. 10.1016/j.meatsci.2015.04.004 25919931

[mbo31157-bib-0053] Wickramasinghe, N. N. , Ravensdale, J. T. , Coorey, R. , Dykes, G. A. , & Scott Chandry, P. (2019). In situ characterisation of biofilms formed by psychrotrophic meat spoilage pseudomonads. Biofouling, 35(8), 840–855. 10.1080/08927014.2019.1669021 31558055

[mbo31157-bib-0054] Zabat, M. A. , Sano, W. H. , Cabral, D. J. , Wurster, J. I. , & Belenky, P. (2018). The impact of vegan production on the kimchi microbiome. Food Microbiology, 74, 171–178. 10.1016/j.fm.2018.04.001 29706333 PMC5965696

[mbo31157-bib-0055] Zeidler, S. , & Muller, V. (2019). Coping with low water activities and osmotic stress in Acinetobacter baumannii: significance, current status and perspectives. Environmental Microbiology, 21(7), 2212–2230. 10.1111/1462-2920.14565 30773801

[mbo31157-bib-0056] Zhang, C. , Alashi, A. M. , Singh, N. , Chelikani, P. , & Aluko, R. E. (2019). Glycated beef protein hydrolysates as sources of bitter taste modifiers. Nutrients, 11(9), 10.3390/nu11092166 PMC6770518 31509959

